# A Functional Bikaverin Biosynthesis Gene Cluster in Rare Strains of *Botrytis cinerea* Is Positively Controlled by *VELVET*

**DOI:** 10.1371/journal.pone.0053729

**Published:** 2013-01-07

**Authors:** Julia Schumacher, Angélique Gautier, Guillaume Morgant, Lena Studt, Paul-Henri Ducrot, Pascal Le Pêcheur, Saad Azeddine, Sabine Fillinger, Pierre Leroux, Bettina Tudzynski, Muriel Viaud

**Affiliations:** 1 Institute for Biology and Biotechnology of Plants, Westfälische Wilhelms-University, Münster, Germany; 2 INRA, UR BIOGER-CPP, Thiverval-Grignon, France; 3 Institute of Food Chemistry, Westfälische Wilhelms-University, Münster, Germany; 4 INRA, IJPB, Versailles, France; Soonchunhyang University, Republic of Korea

## Abstract

The gene cluster responsible for the biosynthesis of the red polyketidic pigment bikaverin has only been characterized in *Fusarium ssp*. so far. Recently, a highly homologous but incomplete and nonfunctional bikaverin cluster has been found in the genome of the unrelated phytopathogenic fungus *Botrytis cinerea*. In this study, we provided evidence that rare *B. cinerea* strains such as 1750 have a complete and functional cluster comprising the six genes orthologous to *Fusarium fujikuroi ffbik1-ffbik6* and do produce bikaverin. Phylogenetic analysis confirmed that the whole cluster was acquired from *Fusarium* through a horizontal gene transfer (HGT). In the bikaverin-nonproducing strain B05.10, the genes encoding bikaverin biosynthesis enzymes are nonfunctional due to deleterious mutations (*bcbik2-3*) or missing (*bcbik1*) but interestingly, the genes encoding the regulatory proteins BcBIK4 and BcBIK5 do not harbor deleterious mutations which suggests that they may still be functional. Heterologous complementation of the *F. fujikuroi* Δ*ffbik4* mutant confirmed that *bcbik4* of strain B05.10 is indeed fully functional. Deletion of *bcvel1* in the pink strain 1750 resulted in loss of bikaverin and overproduction of melanin indicating that the VELVET protein BcVEL1 regulates the biosynthesis of the two pigments in an opposite manner. Although strain 1750 itself expresses a truncated BcVEL1 protein (100 instead of 575 aa) that is nonfunctional with regard to sclerotia formation, virulence and oxalic acid formation, it is sufficient to regulate pigment biosynthesis (bikaverin and melanin) and fenhexamid HydR2 type of resistance. Finally, a genetic cross between strain 1750 and a bikaverin-nonproducing strain sensitive to fenhexamid revealed that the functional bikaverin cluster is genetically linked to the HydR2 locus.

## Introduction

Fungi are an important source of natural bioactive compounds such as antibiotics that may be beneficial for medicine or such as mycotoxins that are problematic in agriculture [1]. The native role of most of these secondary metabolites remains unclear but they may contribute to the fitness of the fungus notably to the protection against biotic and abiotic stresses [2]–[3]. Recent sequencing projects have revealed that fungal genomes may contain up to forty gene clusters for secondary metabolism [4], [5]. Classically, each of these clusters contains the necessary set of genes encoding all enzymes required for the biosynthesis of one compound or multiple structurally closely related compounds. The enzyme responsible for the committed biosynthetic step, often referred to as the “key” enzyme, could be a polyketide synthase (PKS), a non-ribosomal peptide synthetase (NRPS), a hybrid PKS/NRPS, a dimethylallyltryptophan synthase (DMATS), or a terpene synthase. In many cases, the clusters also contain genes encoding specific transcription factors and transporters exporting the metabolites. Clustering of genes involved in the production of one metabolite may provide an evolutionary advantage during horizontal gene transfer (HGT) since it allows the transmission of an entire pathway [6] and the co-regulation of cluster genes by epigenetic regulation mechanisms [7]–[8]. Recently, HGT of several clustered genes has been proven to occur between distantly related fungi [9]–[10] or even from bacteria to ascomycetes [11].

The red pigment bikaverin was first isolated from *Gibberella* (*Fusarium*) *fujikuroi* [12]. As for many secondary metabolites, its production seems to be restricted to closely related species of *Fusarium* [13]: *F. fujikuroi* [14], *F. oxysporum* [15], *F. verticillioides* [16] and *F. proliferatum* [17] while distantly related *Fusarium* species like *F. graminearum* produce another red pigment, aurofusarin [18].

Recently, we identified and characterized the bikaverin biosynthesis gene cluster in *F. fujikuroi* [14]. In addition to the PKS-encoding gene *ffbik1* (for *Fusarium fujikuroi* bikaverin 1, former *ffpks4*; [19]), it contains five genes encoding two biosynthetic enzymes (*ffbik2, ffbik3*), two regulators (*ffbik4*, *ffbik5*), and one transporter (*ffbik6*). The expression of the *ffbik* genes is negatively regulated by high amounts of nitrogen, alkaline pH and by the FfVEL1 protein, a component of the VELVET complex which was recently described as a global regulator of secondary metabolism and differentiation in fungi [8], [14]. In the unrelated fungus *Botrytis cinerea*, known as the gray mold agent, rare strains also produce a red pigment giving a pink coloration to the mycelium [20]–[22]. During monitoring of fungicide resistance, we found two of these pink-pigmented strains additionally being resistant to the fungicide fenhexamid [23]. Recently, the genomes of two gray *B. cinerea* strains, B05.10 and T4, were sequenced, revealing more than forty potential secondary metabolite gene clusters [24] [5]. Interestingly, a nonfunctional bikaverin gene cluster missing the PKS-encoding gene *bik1* was identified [25].

The aim of this study was therefore to verify whether the red pigment produced by *B. cinerea* was bikaverin and to characterize the corresponding biosynthesis genes. We report that the pink *B. cinerea* strains contain the complete functional cluster similar to the one described in *F. fujikuroi*. The results presented highlight the importance of horizontal gene transfer between distantly related fungi for the evolution of fungal secondary metabolism and reveal previously unknown features of the VELVET protein in the regulation of pigment biosynthesis.

## Results

### Only few *Botrytis cinerea* strains possess a complete and functional bikaverin gene cluster

**Figure 1:**
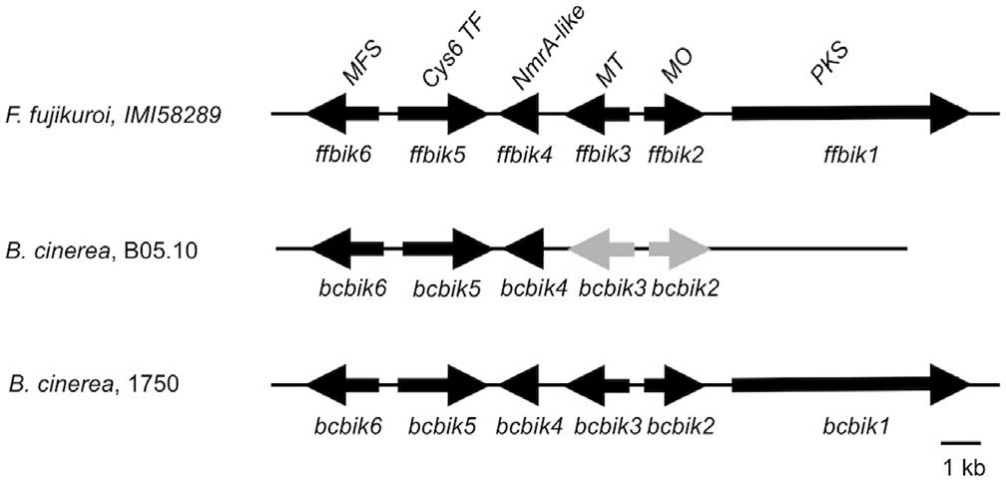
Schematic view of the bikaverin gene clusters in *F. fujikuroi* [**14**], and in gray (B05.10) and pink (1750) *B. cinerea* strains. Complete clusters include a PKS-encoding gene (*bik1*), a putative FAD-dependent monooxygenase (*bik2*), an O-methyltransferase (*bik*3), a putative NmrA-like transcriptional regulator (*bik4*), a Zn(II)_2_Cys_6_ fungal type transcription factor (*bik5*) and an MFS-type transporter (*bik6*).

The bikaverin biosynthesis cluster has only been characterized in *Fusarium fujikuroi* so far [14]. As shown in Figure 1, this cluster contains three biosynthetic genes, *ffbik1, ffbik2* and *ffbik3*, encoding a PKS, a putative FAD-dependent monooxygenase and an O-methyltransferase, respectively. In addition, it includes two genes encoding transcriptional regulators, a putative NmrA-like protein (FfBIK4) and a Zn(II)_2_Cys_6_ transcription factor (FfBIK5), as well as one gene encoding a MFS (major facilitator superfamily)-type transporter (FfBIK6). Recently, an incomplete bikaverin gene cluster, containing the putative orthologues of *ffbik2* to *ffbik6*, was identified in the genomes of the sequenced *B. cinerea* strains B05.10 and T4 [25]. Genes *bcbik2* to *bcbik6* are organized similarly to those in *F. fujikuroi* (Fig. 1), however, an orthologue of *ffbik1* encoding the key enzyme is absent from both sequenced genomes. In addition, many stop mutations and small deletions were identified in *bcbik2* and *bcbik3* when compared to *ffbik2* and *ffbik3* suggesting that they represent pseudogenes [25]. Among B05.10 *bcbik* genes, only *bcbik6* encoding a MFS transporter showed evidence of expression in previous microarray analysis [5]. Taken together, these previously published data suggest that the two sequenced *B. cinerea* strains contain an incomplete and inactive bikaverin gene cluster.

This gene cluster was further investigated in *B. cinerea* strains producing a red pigment. In addition to strains 1750 and 1787 previously reported [23], five other pink strains were collected from different countries and host plants (Table 1). The bikaverin gene cluster was sequenced in three pink strains (1750, 1787 and SEP159) by using primers based on the B05.10 sequence (See Material and methods and Table S1). Sequencing revealed that the organization of *bcbik2* to *bcbik5* is similar to that found in the gray strains (Fig. 1). Because of an AT-rich region (60%) that are present in both gray and pink strains, we failed to sequence the PCR product corresponding to the intergenic region between *bcbik5* and *bcbik6*. However, Southern-blot analysis confirmed that the genes are at the same locus as in the genomes of T4 and B05.10 (data not shown). Interestingly, the 3′ non-coding region of *bcbik2* in the pink strains could not be amplified using primers defined on the B05.10 sequence suggesting a strain-specific polymorphism. A TAIL-PCR approach was therefore initiated by using specific forward primers binding the 3′ coding sequence of *bcbik2* and degenerate primers (See Material and Methods; [26]). By this approach we identified a PKS-encoding gene downstream of *bcbik2* (Fig. 1). In accordance with BIK1 from *F. fujikuroi*, the PKS contains the three essential domains typical of non-reducing PKSs, the ketoacyl synthase (KS), acyl transferase (AT) and phosphopantetheine (PP) domains, as well as an additional thioesterase (TE) release domain [19]. Bidirectional Best Hit analysis (BDBH; [5]) confirmed that this new *B. cinerea* PKS, hereafter called BcBIK1, is the orthologue of *F. fujikuroi* BIK1. Therefore, the bikaverin gene cluster in *B. cinerea* strains 1750, 1787 and SEP159 consists of the same six genes (*bcbik1* to *bcbik6*) and shows the same organization as the bikaverin gene cluster in *F. fujikuroi*. Moreover, the coding regions of the genes in the pink strains do not show any stop codons, frame shifts or deletions suggesting that the expression of these genes lead to functional enzymes and in turn to the biosynthesis of bikaverin. To confirm this hypothesis, the red pigment produced by strains 1750 and 1787 was purified and analyzed by Mass Spectrometry and Nuclear Magnetic Resonance (MS and NMR, see details in Material and Methods). The spectroscopic data were similar to those previously reported for bikaverin [27]–[28] confirming that the gene cluster is functional in the pink strains of *B. cinerea*.

**Table 1:** *Botrytis cinerea* strains used in this study.

Strain and origin	Red Pigment	Fenhe-xamid	Colony diameter on MA, 3 dpi (mm)	Conidia-tion	Sclerotia formation	Lesion diameter on bean, 5 dpi (mm)
B05.10, grape (Germany)	−	S	50.8+/−3.7	+	+	30.8+/−4.7
T4, tomato (France)	−	S	44.8+/−5.8	+	−	18.0+/−6.2
SAS56, grape (Italy)	−	S	52.2+/−6.6	+	+	27.8+/−5.2
SAS405, grape (Italy)	−	S	55.8+/−5.5	+	+	28.9+/−3.7
1750, cucumber (Japan)	+	Hyd R2	45.3+/−8.1	+	−	10.9+/−5.0
1787, strawberry (Japan)	+	Hyd R2	48.7+/−7.6	+	+	32.2+/−4.1
SEP159, tomato (France)	+	S	54.8+/−6.7	+	+	30.3+/−4.8
TM517, almond (USA)	+	S	56.2+/−7.7	+/−	+	No lesion
TM66B02, pistachio (USA)	+	S	53.9+/−6.8	+	+	32.2+/−4.1
TM2884, rose (USA)	+	S	53.5+/−5.6	+	+	32.2+/−4.1
TM2413, grape (NC, USA)	+	S	38.8+/−8.3	+/−	−	No lesion

Fitness characteristics were measured as indicated in Material in Methods.

### The functional bikaverin gene cluster has been acquired through horizontal gene transfer

**Figure 2:**
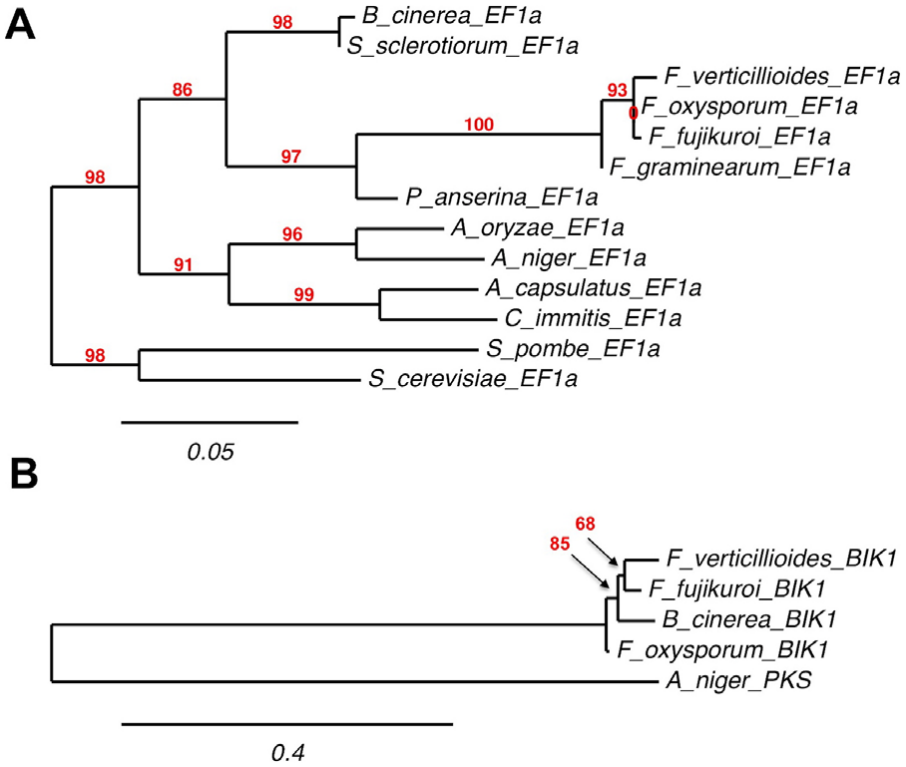
Comparative phylogenies of the conserved elongation factor EF1 α and the BIK1. *Maximum likelihood* phylogenies from alignments of the protein sequences were estimated using the PhyML program with the substitution model WAG and a number of bootstraps of 100 [52] (http://www.phylogeny.fr). A. Phylogeny of EF1α represents the evolution of fungal species. Selected proteins are from *Botrytis cinerea* (BROAD: BC1G_09492.1), *Sclerotinia sclerotiorum* (GenBank: EDO03042.1), *Fusarium verticillioides* (BROAD: FVEG_02381.3), *Fusarium oxysporum* (GenBank: EGU83230.1), *Fusarium fujikuroi* (B. Tudzynski, unpublished data), *Fusarium graminearum* (BROAD: FGSG_08811.3), *Podospora anserina* (NCBI: XP_001907437.1), *Aspergillus oryzae* (GenBank: BAA76296.1), *Aspergillus niger* (NCBI: XP_001398942.1), *Ajellomyces capsulatus* (GenBank: AAB17119.1), *Coccidioides immitis* (GenBank: AAK54650.1), *Schizosaccharomyces pombe* (NCBI: NP_594440.1), *Saccharomyces cerevisiae* (GenBank: AAA34585.1). B. Phylogeny of BIK1 indicates a non-vertical inheritance. Selected proteins are from the pink *B. cinerea* strain 1750 (EMBL: HE802550), *F. fujikuroi* (GenBank: CAB92399.1), *F. oxysporum* (BROAD: FOXG_04757), and *F. verticillioides* (BROAD: FVEG_03379). The PKS sequence from *A. niger* (JGI: ANG51499) was used to root the tree.

The recent phylogenetic study of Campbell et al. [25] revealed that *B. cinerea* strains B05.10 and T4 have inherited *bcbik2* to *bcbik6* from *Fusarium* through an HGT. Because of the lack of *bcbik1* in these gray strains, it was not known whether the complete bikaverin gene cluster was initially transferred between the two distantly related species. The identification of the complete and functional bikaverin cluster in few *B. cinerea* strains provided the opportunity to answer this question. We performed a phylogenetic analysis on the basis of the six *bcbik* functional genes of strain 1750. The distribution of the bikaverin gene cluster among fungal genomes was investigated by similarity searches using the *F. fujikuroi* FfBIK protein sequences [14] (Material and methods). Corresponding genes of *F. oxysporum* and *F. verticillioides* were found in the genome database at the Broad Institute (http://www.broadinstitute.org/scientific-community/science/projects/fungal-genome-initiative/fungal-genome-initiative). In these two bikaverin-producing species the cluster organization is identical to that described in the closely related species *F. fujikuroi* [14] [25]. From the current genomic data, *B. cinerea* appears to be the only species sharing the bikaverin cluster with the *Fusarium* species as the cluster could not be detected in any other fungal species sequenced so far. The amino acid sequences of BcBIK1 to BcBIK6 of the pink strain 1750 were aligned with the proteins from the *Fusarium* species and maximum likelihood phylogenies were estimated. Figure 2 presents the phylogenetic tree of the longer and more polymorphic BIK1 compared to the tree of the conserved elongation factor EF1α whose evolution represents the evolution of fungal species. According to the recent phylogenetic study of Watanabe et al. [29], the bikaverin-producing *Fusarium* species are closely related as they all belong to the Clade V of the *Fusarium* genus. Inside this clade, *F. verticillioides* and *F. fujikuroi* are members of the *G. fujikuroi* species complex (also named *Liseola* section) while *F. oxysporum* stands apart (in the *Elegans* section). The phylogenetic tree shows that the evolution of BIK1 from the *Fusaria* fits with the evolution of these species, suggesting that BIK1 was vertically inherited from a common ancestor. In contrast, the position of BcBIK1 inside the *Fusarium* branch is not compatible with a vertical inheritance of the gene. Phylogenetic analysis of BIK2 to BIK5 also placed the *B. cinerea* proteins inside the group of *Fusarium* proteins. Nevertheless, the bootstrap values on the branches were weaker than for BIK1, probably because of the smaller number of informative polymorphic amino acids compared to the PKS (Fig. S1). Finally, the protein sequences were concatenated to generate a tree based on the polymorphism of the whole set of BIK proteins that resembles the BIK1 tree (Fig. S1). In sum, these data demonstrate that *B. cinerea* has acquired the functional bikaverin gene cluster from *Fusarium* through a HGT.

To get an indication of how widely distributed the bikaverin cluster in the genus *Botrytis* is, we investigated its presence in 30 strains of *B. cinerea* and 15 other *Botrytis* species that belong either to the clade 1 or to the clade 2 defined by Staats et al. [30] (Table S2). *Bcbik6* was detected by diagnostic PCR in all *B. cinerea* strains, as well as in the species *B. pelargonii* and *B. pseudocinerea* that are members of clade 1 like *B. cinerea* [31], and in *B. tulipae* belonging to clade 2 [30]. By contrast, the PKS-encoding gene *bcbik1* was only found in the seven *B. cinerea* strains that produce the red pigment. The distributional patterns of the bikaverin gene cluster within the genus *Botrytis* suggest that the HGT probably occurred from *Fusarium* to a common ancestor of *Botrytis* clade 1 and 2 and that most of the strains of the investigated species have lost either the whole or a part (*bcbik1*) of the bikaverin cluster later on rendering them incapable to produce bikaverin.

### Bikaverin-producing strains are heterogenous with regard to differentiation and virulence

The seven pink *B. cinerea* strains collected so far (Table 1) do not share any common geographic origin or host. In order to investigate whether the production of bikaverin affects differentiation and virulence in *B. cinerea*, several fitness characteristics of pink strains were compared to those of gray strains (B05.10, T4, SAS405 and SAS56). Radial growth rates on different solid media such as on minimal (MM) and rich medium (MA), varying light conditions (16 h light per day *versus* complete dark), and different temperatures (18, 23 and 27°C; See Material and Methods) gave similar results (Table 1; data not shown). On all tested media, the pink strains conidiated like the gray strains, with the exception of TM517 and TM2413 that only produce very few conidia. Formation of sclerotia in darkness occurred for all strains except 1750, TM2413 and T4. Virulence tests also revealed heterogeneity among the pink strains with two strains being nonpathogenic (TM517 and TM2413) on bean leaves and one strain (1750) exhibiting reduced virulence as the gray strain T4. In conclusion, no common biological feature could be identified among the bikaverin-producing strains when compared to the gray reference strains indicating that bikaverin production does not affect processes such as light-dependent differentiation and virulence.

### BcBIK4 of the bikaverin-nonproducing strain B05.10 is fully functional

**Figure 3:**
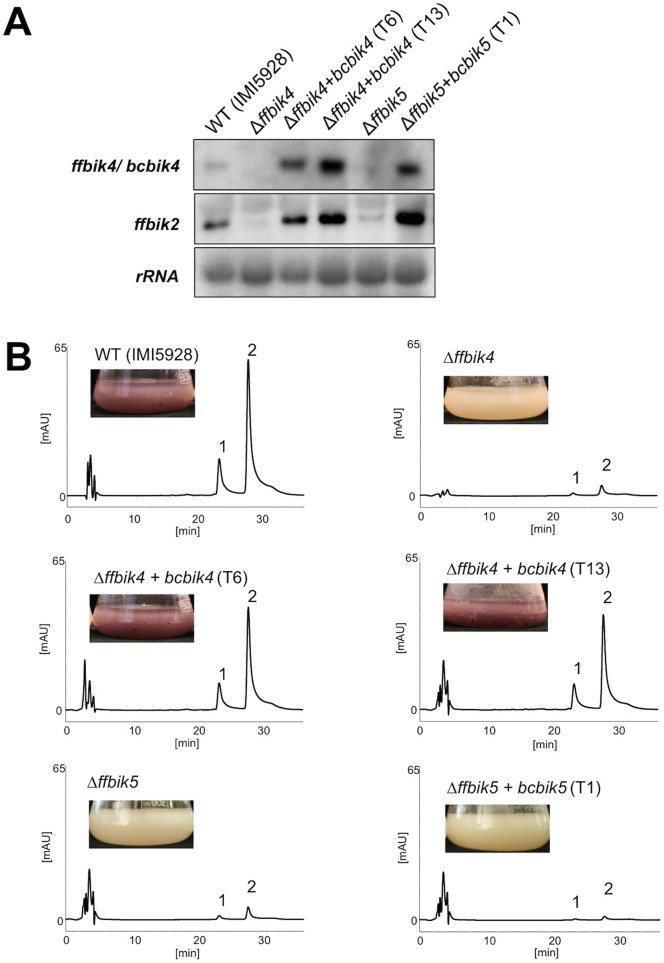
Heterologous expression of *bcbik4* and *bcbik5* of the gray strain B05.10 in the respective *Fusarium fujikuroi* (IMI58289) deletion mutants. A. Expression of *ffbik2* and *ffbik4/bcbik4.* Wild type (IMI58289) and mutants were grown for three days in 10% liquid ICI (0.6 mM glutamine) medium at 28°C and 180 rpm. The northern blots were hybridized with probes for *ffbik2* or simultaneously hybridized with probes for *ffbik4* and *bcbik4*. rRNA is shown as loading control. B. Identification of bikaverin and norbikaverin production. Wild type (IMI58289) and mutants were grown in liquid ICI medium containing 6 mM glutamine for three days. Samples were taken and used for HPLC-DAD analyses (detected at 450 nm) as described in Material and Methods. Peaks for norbikaverin (1) and bikaverin (2) are labeled in the chromatograms.

Based on bioinformatics analyses of the evolution of the incomplete bikaverin gene clusters found in strains B05.10 and T4, Campbell et al. [25] suggested that the regulatory genes *bcbik4* and *bcbik5* encoding a NMR-like protein and the putative pathway-specific Zn(II)_2_Cys_6_ transcription factor, respectively, are probably functional. They postulated selective preservation of these regulatory genes, and especially of *bcbik4*, due to its possible involvement in other non-bikaverin-related biosynthetic pathways in *B. cinerea*. To verify this hypothesis experimentally, we expressed *bcbik4* and *bcbik5* from the gray strain B05.10 driven by the *A. nidulans oliC* promoter in *F. fujikuroi* Δ*ffbik4* and Δ*ffbik5* mutants, respectively. In Δ*ffbik4* and Δ*ffbik5* mutants, bikaverin production is drastically reduced due to the down-regulation of the three biosynthetic genes, *ffbik1-ffbik3* [14]. Transformants overexpressing *bcbik4* (Δ*ffbik4+bcbik4*) showed the expected strong expression of *bcbik4* (Fig. 3A) and full restoration of bikaverin production as shown by HPLC spectra (Fig. 3B). On the other hand, Δ*ffbik5* mutants expressing *bcbik5* (Δ*ffbik5+bcbik5*) did not produce the pigment. However, the BcBIK5 transcription factor must be partially functional, since it restores the expression of *ffbik4* and *ffbik2* (Fig. 3A). In summary, BcBIK4 and BcBIK5 of the bikaverin-nonproducing strain B05.10 retained full or partial functionality and were able to restore expression of other cluster genes (BcBIK4 and BcBIK5) and bikaverin biosynthesis (BcBIK4).

### Bikaverin formation in strain 1750 is regulated by the VELVET protein BcVEL1

The VELVET protein complex is known for its function in coordinating secondary metabolism and light-dependent development in filamentous fungi (reviewed in [8]), and members of this protein complex were previously shown to regulate the biosynthesis of bikaverin and other secondary metabolites in *F. fujikuroi* [14]. Recently, we identified the VeA/FfVEL1-homologous gene of *B. cinerea* (*bcvel1*) by a map-based cloning approach for unraveling the genetic basis for the phenotypical differences between the two sequenced *B. cinerea* strains: the virulent strain B05.10 and the less virulent strain T4. We demonstrated that a single nucleotide polymorphism (SNP) in the T4 *bcvel1* homologue (*bcvel1^T4^)* leads to a truncated and nonfunctional BcVEL1 protein. Thus, the replacement of the truncated *bcvel1^T4^* copy by the native copy (*bcvel1^B05.10^*) in the T4 genomic background resulted in the B05.10 phenotype and in turn, the deletion of *bcvel1* in B05.10 (B05.10:Δ*bcvel1*) resulted in the T4 phenotype. T4 and B05.10:Δ*bcvel1* mutants are less virulent on bean plants, do not produce oxalic acid and form abundant conidia instead of sclerotia in continuous darkness. Notably, the production of the phytotoxic secondary metabolites botrydial and botcinic acid is not affected by the *bcvel1* mutations. Vegetative growth of the deletion mutant, however, was accompanied by increased secretion of the pigment melanin resulting in dark-colored culture broths [32].

**Figure 4:**
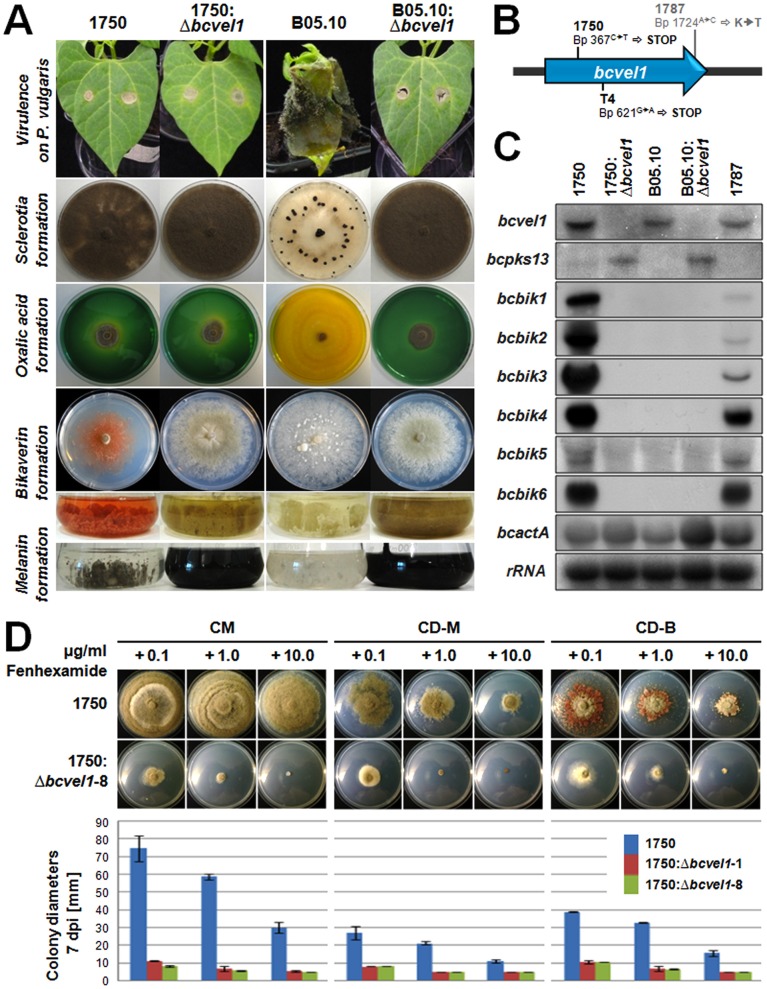
Role of the global regulator BcVEL1 in the pink strain 1750. A. Phenotypes caused by the deletion of *bcvel1* in two different genetic backgrounds, 1750 and B05.10. Virulence of strains was monitored on living plants of *Phaseolus vulgaris* (7 days after inoculation), sclerotial development by incubation on complete medium (CM) in continuous darkness (14 days), oxalic acid production by using the pH indicator bromothymol blue (color change from green to yellow indicate the acidification of the culture medium) (7 days), bikaverin formation by incubation in liquid or on solid CD-B medium in the dark (7 days), and melanin formation by incubation in liquid CD-M medium in light-dark conditions (7 days). B. Sequencing of *bcvel1* in the pink strains 1750 and 1787 revealed several SNPs. The point mutation of base pair 367 in 1750 results in a stop codon; a similar mutation leading to a truncated BcVEL1 was recently found in strain T4 [32]. Both 1750 and 1787 comprise additional point mutations that are either silent or result in amino acid exchanges (see also Fig. S2). C. Detection of *bcbik1-6 expression in pink (1750, 1787) and gray (B05.10) strains.* Strains were grown for 3 days on solid CD-B medium covered with cellophane overlays. Northern blots were hybridized with probes for *bcvel1*, *bcpks13* (encoding the polyketide synthase that catalyzes the first step in melanin biosynthesis), *bcbik1-6*, and *bcactA* (actin). rRNA is shown as loading control. D. Effect of fenhexamid on growth rates of 1750 and 1750: Δ*bcvel1* mutants. Strains were grown on complete medium (CM), CD-M (bikaverin-non-inducing conditions) or CD-B (bikaverin-inducing conditions) supplemented with the indicated concentrations of fenhexamid. Diameters of six colonies per strain and condition were measured after 7 days of incubation; pictures were taken after 13 days.

To investigate if BcVEL1 regulates the formation of the red pigment bikaverin as well, we deleted the gene in the bikaverin-producing strain 1750. In two independent transformants (designated as 1750: Δ*bcvel1*-T1, and -T8) *bcvel1* was replaced by the hygromycin resistance cassette. In the first instance, we studied the impact of the *bcvel1* deletion on bikaverin formation. Strain 1750 produces bikaverin when cultivated in modified Czapek-Dox medium containing ammonium nitrate instead of sodium nitrate as nitrogen source (CD-B) under dark conditions (Fig. 4A). In contrast, 1750: Δ*bcvel1* mutants failed to produce bikaverin and did not express the cluster genes (*bcbik1-bcbik6*) indicating that BcVEL1 functions as a positive regulator of bikaverin formation (Fig. 4A).

We also searched for phenotypes that were associated with the *bcvel1* deletion in B05.10, such as reduced virulence, loss of sclerotia and oxalic acid formation, and overproduction of melanin. Strikingly, strain 1750 exhibited some of the characteristics of strain T4 expressing a mutated BcVEL1 and Δ*bcvel1* mutants derived from strains 1750 and B05.10; i.e. all four strains formed conidia instead of sclerotia during incubation in continuous darkness, exhibited reduced virulence on bean leaves, and did not produce oxalic acid. This indicates that strain 1750 itself may represent a natural *bcvel1* mutant as it was previously described for strain T4. Sequencing of the *bcvel1* open reading frame in 1750 revealed several SNPs involving base pairs 167, 234, 367, 981, 1080, and 1724 (Fig. S2). All SNPs except for that found at base pair 367 are also present in *bcvel1* of the pink strain 1787 indicating a closer relationship of both bikaverin producers with each other than with the gray strains (e.g. B05.10 and T4). Most mutations caused by the common SNPs in 1750 and 1787 are silent, the SNP at bp 1724 results in an amino exchange. The SNP at bp 367 (C to T) that is unique to strain 1750, however, introduces a stop codon and consequently leads to a shorter protein of only 100 aa (BcVEL1^1750^) instead of 575 aa (wild-type protein: BcVEL1^B05.10^). Therefore, phenotypes of 1750 concerning virulence, sclerotial development/light-independent conidiation, and oxalic acid formation can be assigned to the identified SNP in 1750. However, the phenotype of 1750: Δ*bcvel1* mutants differed from that of 1750 expressing the truncated BcVEL1 protein with regard to pigment formation: firstly, the deletion mutant lost the ability to produce bikaverin, and secondly, the deletion mutant but not 1750 exhibited increased melanin formation (Fig. 4C). Based on this fact, we conclude that the first 100 aa of the BcVEL1 protein are sufficient for regulation of bikaverin and melanin formation while the regulation of light-dependent differentiation and virulence requires the full-length protein.

Aside from the production of bikaverin, the strains 1750 and 1787 share an uncommon phenotype i.e. a specific type of resistance towards the fungicide fenhexamid (HydR2; [23]; Table 1). Having a 1750 mutant in hand that lacks the expression of *bcbik1* to *bcbik6* and consequently the formation of bikaverin, we studied the relationship between bikaverin and fenhexamid resistance by performing growth assays on solid media differentially affecting bikaverin formation (CM and CD containing sodium nitrate or ammonium nitrate as single nitrogen source) that were supplemented with different concentrations of fenhexamid. As shown in Fig. 4D, the lack of bikaverin gene cluster activity in 1750: Δ*bcvel1* mutants was accompanied by the inability of the mutant to grow on medium containing fenhexamid, indicating that BcVEL1 regulates both characteristics. However, the loss of resistance is likely not correlated with the loss of the pink pigmentation of the mycelium, as 1750 is resistant to fenhexamid even on media that do not stimulate bikaverin production (CM and CD-M).

### The bikaverin gene cluster of strain 1750 is genetically linked to the HydR2 locus responsible for a specific type of fenhexamid resistance

A formal genetic analysis was employed to test whether there is a link between the functional bikaverin cluster and the HydR2 type of fenhexamid resistance. A cross between the HydR2 pink strain 1750 and a gray strain sensitive to fenhexamid (HydS) was realized. Microconidia from 1750 were mixed with sclerotia of the two reference strains SAS56 and SAS405 exhibiting the *MAT1-1* and *MAT1-2* mating types, respectively [33]. Apothecia were recovered from the fertilization of sclerotia obtained from SAS405 with microconidia of 1750 indicating that 1750 possesses the MAT1-1 mating type. Seventy-three single ascospore-derived progeny were analyzed for their capability to produce bikaverin (Table S3). About half (40) of them produced pink mycelia when grown on solid YSS medium suggesting that only one locus is responsible for the phenotype. PCR tests furthermore showed that the individuals able to produce the pink pigment contain *bcbik1*, and in turn that gray individuals lack *bcbik1*. These data confirm that the described gene cluster is responsible for the production of bikaverin. PCR analyses using specific primers for detection of the two mating types (MAT1-1 or MAT1-2) confirmed that genetic recombination between both parental strains had taken place; both mating types were found although not at equal ratios among the pink and gray individuals. Finally, we evaluated the linkage between bikaverin biosynthesis and fungicide resistance by cultivating the individuals on fenhexamid-containing media. All pink individuals showed the HydR2 phenotype as the parental strain 1750 while all except for one individual of the gray progeny were sensitive to fenhexamid resembling the HydS phenotype of parental strain SAS405 (number 52, Table S3). These segregation data suggest that the bikaverin cluster is genetically linked to the locus responsible of the HydR2 type of resistance in strain 1750.

## Discussion

The recent sequencing of ascomyceteous genomes has revealed the large number and diversity of secondary metabolic gene clusters [3]. For instance, the genome of *B. cinerea* contains more than 43 potential secondary metabolism gene clusters; only 19 of them are shared with the closely related species *Sclerotinia sclerotiorum* although most genes have orthologues in at least one other more distantly related fungus [5]. This “patchy” distribution of secondary metabolism gene clusters in fungal species may be due to gene losses, but also due to acquisition of new genes by HGT between species. Such HGTs are assumed when clusters present in distantly related species show high degrees of microsynteny and when the orthologues exhibit high levels of amino acid identity [10] [9]. The hypothesis of a HGT from a donor species to a receptor species could be validated by phylogenetic analysis *i.e*. the genes of the receptor species are expected to group with those of the donor species in gene phylogenies and to be in stark contrast to the host species phylogeny. Using these approaches, a couple of HGTs between unrelated fungal species have recently been demonstrated. A cluster of five to six genes including a PKS-NRPS-encoding gene was shown to be transferred from a donor close to *Magnaporthe grisea* to an ancestor of *Aspergillus clavatus* [9]. More recently, Slot and Rokas [10] provided evidence that the complete and functional sterigmatocystin biosynthesis gene cluster that is more than 50 kb in size was transferred from *Aspergillus* to *Podospora anserina*. Finally, Campell *et al*. [25] have shown that the sequenced genomes of the two *B. cinerea* strains T4 and B05.10 contain a nonfunctional bikaverin gene cluster that was acquired through HGT from a *Fusarium* ancestor. Because of the degenerated biosynthesis genes (*bcbik 2* and *bcbik 3*) and the absence of *bik1* encoding the key enzyme PKS, they postulated that the remaining *bcbik* genes were part of a dead bikaverin gene cluster. Our work provides evidence that this HGT originally also included the gene encoding the PKS (BIK1), and that the complete cluster is still present in strains of *B. cinerea* with the pink-mycelium phenotype, which are a minority of strains of this species. Indeed, phylogenetic analysis clearly showed that BcBIK1 nests within the *Fusarium* BIK1 proteins demonstrating that the gene was acquired horizontally from *Fusarium*. The facts that the *bcbik1 to bcbik-6* genes sequenced in the pink strains do not show any deleterious mutation as the genes of the gray strains T4 and B05.10 do, that the cluster comprising *bcbik1* to *bcbik6* co-segregates with pigment production in a genetic cross between a pink and a gray strain, and that MS and NMR analysis identified the produced pink pigment as bikaverin demonstrate that the cluster is fully functional in few *B. cinerea* strains giving them the capability to form bikaverin The presence of *bcbik6* in other *Botrytis* species (*B. pseudocinerea, B. pelargonii* and *B. tulipae*) indicates that the HGT occurred in an ancestor of *B. cinerea* before the divergence of the clade 1 and 2 [30]. However, our PCR screen did not detect a complete cluster in any of these other species suggesting that in most cases, the ability to produce bikaverin was lost.

The production of bikaverin by only a minority of *B. cinerea* strains raises the question of the biological function of this compound in the life cycle of the gray mold fungus. The acquisition and the conservation of the ability to produce a new secondary metabolite is likely driven by the need to survive in a particular environmental niche [2]–[3]. In the case of bikaverin in *B. cinerea*, this niche could not be anticipated from the origin of the seven known pink strains since they were collected from different continents and host plants. In controlled growth conditions, the pink strains showed heterogeneous behaviors in developmental processes such as formation of conidia and sclerotia. Furthermore, our experiments did not reveal any particular adaptation to temperature variations or light conditions when compared to the gray strains. Finally, virulence on bean leaves also illustrated the heterogeneity of the strains, with two of them being nonpathogenic.

Fungi are known to produce several pigments, in most cases PKS-derived secondary metabolites [34]. *B. cinerea* and many other fungi produce dihydroxynaphthalene (DHN)-melanin that is excreted to the cell wall to protect survival structures from UV radiation, oxidizing agents, and desiccation [35]. Melanin is found in both macroconidia and sclerotia that are produced by *B. cinerea* depending on the environmental light conditions [36]–[37]. Melanization of sclerotia may protect them from mycoparasitism [38]. DHN-melanin biosynthesis involves a non-reducing PKS enzyme of clade II as defined by Kroken et al. [24]. The genome of *B. cinerea* contains a gene encoding such a PKS (*bcpks13*), clustered with two other genes that could contribute to melanin biosynthesis [5]. In contrast, *Fusarium* species including the bikaverin-producing species *F. verticillioides, F. oxysporum*, and *F. fujikuroi*, lack the PKS-encoding gene responsible for DHN-melanin biosynthesis [24]. In these species, the production of bikaverin could be an alternative way to protect the fungus from UV damages. Recently, a second PKS-derived red pigment, the perithecia pigment fusarubin, has been identified in *F. fujikuroi* which was shown to mechanically stabilize the fruiting bodies and probably also protects these structures from environmental stresses [39]. Whether the production of both melanin and bikaverin by some *B. cinerea* strains provides an increased protection against UV light remain to be investigated. Bikaverin was also previously described as an antibiotic effective against protozoa, ascomycetes and oomycetes [13] [15]. Therefore, it may be involved in inhibition of competing microorganisms that interact with *Fusarium spp*. and *B. cinerea*.

The gray strains of *B. cinerea* are impaired in bikaverin production because of the absence or deleterious mutations in the biosynthetic genes (*bcbik1-3*) but the previous sequence analysis of the regulatory genes *bcbik4* and *bcbik5* suggested that they are still functional [25]. This was confirmed by our heterologous complementation approach: *bcbik4* and *bcbik5* from the gray strain B05.10 were shown to fully or partially complement the corresponding deletion mutants of *F. fujikuroi* [14]. While BcBIK4 restored bikaverin production in the Δ*ffbik4* mutant, BcBIK5 was able to overcome the down-regulation of bikaverin cluster genes, e.g. *ffbik4* and *ffbik2*, in the Δ*ffbik5* mutant. The reason why overexpression of *bcbik5* did not restore bikaverin production but only expression of cluster genes might be the partial deletion of the adjacent *ffbik6* gene in the Δ*ffbik5* mutant due to the *ffbik5* gene replacement strategy used [14]. The fact that the regulatory genes *bcbik4* and *bcbik5* are still functional despite the absence of functional biosynthesis genes (*bcbik1-3*) suggest that they may have acquired some additional regulatory functions in the expression of *B. cinerea* genes unrelated to the production of the red pigment as hypothesized by Campbell et al [25].

The work presented here has also explored the upstream regulation of the bikaverin cluster in *B. cinerea*. Expression of the gene cluster and the production of bikaverin depends on the global regulator VELVET (BcVEL1), as the deletion of *bcvel1* in a pink strain resulted in the absence of *bcbik1-6* expression and consequently in nonpigmented mutants. However, in contrast to the situation in *F. fujikuroi*, VEL1 acts as positive regulator of bikaverin formation in *B. cinerea*. The significant up-regulation of bikaverin formation in Δ*ffvel1* mutants is restored to wild-type levels by heterologous expression of BcVEL1 [32] suggesting that the antagonistic regulation of bikaverin formation by VELVET depends on the fungus and its downstream signaling processes rather than on the VEL1 protein. VELVET (VeA) homologues are known for their role in coordinating light-dependent development and secondary metabolism in ascomycetes, thereby positively or negatively affecting secondary metabolism, e.g. production of toxins and pigments [8]. Thus, FfVEL1 is a repressor of bikaverin formation and at the same time a positive regulator of the production of gibberellic acids, fumonisins and fusarin C [14]. Interestingly, and in contrast to many other fungi, BcVEL1 does not affect the formation of the two most prominent secondary metabolites (botrydial and botcinic acid). Nevertheless, *bcvel1* mutants revealed significantly reduced virulence [32]. Similarly to *B. cinerea*, the deletion of VeA homologues affects pigment formation in other fungi, e.g. causing excessive brown pigmentation in *Aspergillus nidulans* [40], increased DHN melanin biosynthesis in *Cochliobolus heterostrophus* [41], and loss of aurofusarin formation in *F. graminearium* [42]–[43], of melanin formation in *Mycosphaerella graminicola* [44], and of the yellow pigmentation in *Trichoderma virens* [45].

By characterizing the pink strain (1750) that we chose for the *bcvel1* knock-out approach we identified a SNP in *bcvel1* leading to a truncated protein. Therefore, strain 1750 exhibits the “*velvet* phenotype” (light-independent conidiation, loss of sclerotia formation and oxalic acid production, reduced virulence) as previously described for the *B. cinerea* strain T4 and the *bcvel1* deletion mutant in the B05.10 background [32]. Both pink strains 1787 and 1750 share several SNPs in *bcvel1*; the only exception is the SNP that leads to the stop codon in 1750. Whether the other pink strains contain SNPs in their *bcvel1* homologues as well needs to be investigated. However, as most of them are virulent and able to produce sclerotia, it can be ruled out that they are sharing the detrimental SNP with strain 1750. SNPs in the *bcvel1* are likely quite common, and so far two different loss-of-function mutations were described resulting in a 100-aa protein in the bikaverin producer 1750 and in a 184-aa protein in the nonproducing strain T4. Due to the fact that the phenotype of Δ*bcvel1* mutants differed from that of the progenitor 1750, different functions can be attributed to the N- and C-terminal parts of the VEL1 protein in *B. cinerea*. Based on the previous results, the presence of the C terminus (aa 185–575) of BcVEL1 is a prerequisite for nuclear localization, sclerotia formation, oxalic acid formation and full virulence [32] while the data obtained from the current study provide evidence that only the first 100 aa of BcVEL1 are required for regulation of pigment formation (repression of melanin formation and stimulation of bikaverin formation, respectively). Considering the fact that the expression of the truncated BcVEL1 protein in 1750 does not represent a loss-of-function mutation of BcVEL1 with regard to pigment formation, we compared the growth characteristics of both strains 1750 and T4. Like 1750, strain T4 did not show an increase in melanin production. Furthermore, both strains displayed reduced growth rates on standard media and formed conidia instead of sclerotia in constant darkness, while they produced fewer conidia compared to the wild type B05.10 and the respective Δ*bcvel1* mutant (data not shown). To gain a better understanding of the signaling function of BcVEL1 it will be necessary to investigate other proteins of the VELVET complex such as the homologues of *A. nidulans* VelB, VosA and LaeA. For instance, it was shown in *A. nidulans* that VeA interacts via the N terminus with VelB in the cytoplasm, and via the C terminus with the putative histone methyltransferase LaeA after entry of the VeA-VelB dimer into the nucleus [40].

Two of the studied pink strains show a HydR2 type of resistance to the hydroxyanilide fenhexamid, a botryticide whose target site is the 3-ketoreductase involved in the sterol C4 demethylation process [46]. Until now, three main phenotypes exhibiting specific resistance to fenhexamid have been identified among gray mold populations. HydR1 corresponds to the naturally resistant species *B. pseudocinerea* recently described [31]. In this species, resistance to fenhexamid is mainly determined by detoxification of the hydroxyanilide by a dedicated cytochrome P450 monooxygenase (A. Billard et al., unpublished). In *B. cinerea*, two types of resistance have been characterized. HydR3 corresponds to qualitative changes in the sterol 3-ketoreductase, the target of fenhexamid [47]–[48]. The mechanism of resistance in HydR2 strains remains unknown but the involvement of a yet unidentified monooxygenase, different from that of HydR1 strains, leading to an increase detoxification of fenhexamid has been suggested in both gray and pink strains [23]. The phenotype of 1750:Δ*bcvel1* mutants revealed that the *hydR2* locus is positively regulated by the VELVET complex similar to the bikaverin cluster. In addition, our analysis of a genetic cross between the HydR2 pink strain 1750 and a gray HydS strain suggested that the *hydR2* locus is genetically linked to the bikaverin cluster because both characters co-segregated in all but one ascospore isolates. Nevertheless, the presence of one gray resistant recombinant furthermore indicated that the complete gene cluster is not required for the HydR2 phenotype. At this stage, our hypothesis is that the *hydR2* locus is either the *bcbik2* gene encoding a FAD-dependent monooxygenase, either another gene encoding a monooxygenase that is located near to the bikaverin gene cluster. Further genetic analyses are needed to identify this locus and the associated enzymatic activity involved in fenhexamid detoxification.

In summary, the existence of the complete bikaverin biosynthesis pathway in *B. cinerea* reveals that HGT contributed to the evolution of secondary metabolism and to the diversity of the gray mold populations. In *B. cinerea*, the bikaverin cluster is positively regulated by the VELVET protein BcVEL1 while the original cluster in *Fusarium* is negatively regulated by FfVEL1. Finally, the identification of another natural mutation of *bcvel1* in strain 1750 highlights this gene as an important source for achieving phenotypic variation, e.g. with regard to light-dependent differentiation, virulence and pigment formation.

## Materials and Methods

### Strains and culture conditions

The strains of *Botrytis cinerea* Pers.: Fr. [*Botryotinia fuckeliana* (de Bary) Whetz] used in this study are listed in Tables 1 and S2. All newly isolated strains were genetically purified by generating cultures from single conidia. The genomes sequences of *B. cinerea* B05.10 and T4 strains are publicly available (strain T4: http://urgi.versailles.inra.fr/index.php/urgi/Species/Botrytis; strain B05.10: http://www.broadinstitute.org/annotation/genome/botrytis_cinerea/). Standard procedures for culturing and maintaining of *B. cinerea* strains and mutants were carried out at 21°C with 16 h daylight per day whereas sclerotia formation was induced by culturing the strains in complete darkness. Cultures were done on malt agar medium (MA; 2 g l^−1^ malt extract, 2 g l^−1^ yeast extract, 15 g l^−1^ agar), and minimal medium (MM; 10 g l^−1^ glucose, 2 g l^−1^ NaNO_3_, 500 mg l^−1^ KH_2_PO_4_, 250 mg l^−1^ MgSO_4_, 7H_2_O, 250 mg l^−1^ KCl, 1 mg l^−1^ FeSO_4_, 7H_2_O, 469 mg l^−1^guanine, 15 g l^−1^ agar). Estimation of growth rates and conidiation was examined on three different plates at 3, 6 and 10 days post-inoculation. Sensitivity to fenhexamid was established on mycelial growth on solid YSS medium (2 g L^−1^ of yeast extract, 10 g L^−1^ of glucose, 2 g L^−1^ of KH_2_PO_4_, 1.5 g L^−1^ of K_2_HPO_4_, 1 g L^−1^ of (NH_4_)_2_SO_4_, 0.5 g L^−1^ of MgSO_4_ 7H_2_O) supplemented with fenhexamid (technical grade, kindly provided by Bayer CropScience SAS) at 0.5, 2 and 5 µg ml^−1^. Plates were inoculated with 3 day-old unsporulated mycelial plugs and incubated at 20°C in the dark during 3–4 days. Resistant strains produced mycelium at fenhexamid concentrations up to 5 µg ml^−1^, low resistant strains up to 2 µg ml^−1^. Sensitive strains did not grow at these concentrations.

### Identification and sequencing of the bikaverin gene cluster

Genomic DNA was extracted from fungal mycelium using a Sarcosyl-based protocol [49]. PCR reactions were done with the Silverstar *Taq* DNA polymerase (Eurogentec) except for the sequencing for which the *Pfu* high fidelity polymerase (Fermentas) was used. Gel electrophoresis, restriction enzyme digestion, Southern experiments were performed using standard protocols [50]. DNA probes were labeled by the random primer method using the ready-to-go DNA Labeling beads (Amersham) and 20 μCi α-32P-dCTP. The bikaverin clusters of the pink strains 1750, 1787 and SEP159 were sequenced by designing PCR primers on basis of the B05.10 sequence (BROAD references BC1G_15238.1 to 15242.1; primers in Table S1). Seven overlapping PCR products ranging from 1.4 kb to 5.3 kb were obtained. These fragments were purified with PEG8000 solution (26.2% polyethylene glycol 8000, 6.6 mM MgCl_2_ and 0.6 M NaOAc) and double-strand sequenced at the INRA-BIOGER genotyping platform using a CEQ8000 capillary sequencing system (Beckman Coulter). In order to investigate the genomic region downstream of *bcbik2*, several rounds of TAIL (thermal asymmetric interlaced)-PCR were performed as described in Liu et al. [26]. The designed trios of successive forward primers are indicated in Table S1. Sequences of *bcbik1* to *bcbik6* of the strain 1750 have been deposited at EMBL (accession numbers HE802545 to HE802550).

### Chemical identification of bikaverin

*B. cinerea* strains 1750 and 1787 were grown in liquid malt medium for one week. The red pigment was extracted with dichloromethane and purified using TLC. MS using direct injection, electrospray ionization and positive detection allowed the attribution of a molecular weight of 382 through observation of both [M]^+^ (m/z 382) and [M+Na]^+^ (m/z 425) ions. ^1^H NMR spectrum (300 MHz in CDCl_3_) revealed the highly substituted aromatic structure of this compound through the observation of only three aromatic resonances at respectively 6.39 (s), 6,8 (bs) and 6.97 (bs) ppm but also the presence of three methoxy groups, two of which corresponding to methoxy groups (3.92 and 3.96 ppm), the third one being characteristic of a methyl linked to an aromatic ring (2.85 ppm). Due to the low quantity of compound, only partial ^13^C data were obtained (75.5 MHz in CDCl_3_) where some resonances of quaternary carbons were missing. Indeed, only three resonances were observed in the region or aromatic carbons bearing phenolic groups (164.5, 159.1 and 158.5 ppm) and only one in the carbonyl region (180.4 ppm). Nevertheless, resonances were also observed for the three methyl groups (56.8, 56.0 and 23.4 ppm) and three methine aromatic carbon (99.0, 112.5 and 117.9 ppm). Out of this data, the characteristic chemical shifts of 6.39 ppm for the proton and 98.9 ppm for the corresponding carbon were consistently in agreement for the presence of the peculiar 2-methoxy 1,4-*p*-quinonic moiety of bikaverin. These spectroscopic data are in good agreement with those of the already reported data of bikaverin [27]–[28].

For bikaverin analysis of two day-old cultures of *F. fujikuroi*, the culture fluids were filtered over a 0.2 µm-pore-size membrane filter (Millex; Millipore) and measured directly using high performance liquid chromatography coupled with a diode array detector (HPLC-DAD) as previously described [39].

### Phylogenetic analysis

Genes responsible for bikaverin biosynthesis in *F. fujikuroi* [14] were used to search for orthologues in fungal genomes available at the NCBI (http://blast.ncbi.nlm.nih.gov/Blast.cgi) or at the BROAD institute (http://www.broadinstitute.org). For this purpose, similarity searches were run using the FfBIK proteins and the BLAST program. Orthology links were confirmed by using the orthoMCL version 1.4 program [51]. Protein sequences were aligned with the MUSCLE program, alignments were curated with the Gblock program, phylogenetic analyses were performed using the PhyML program and resulting tree were obtained with the TreeDyn program [52] (http://www.phylogeny.fr).

### Sexual crosses and segregation analysis

Sexual crosses between *B. cinerea* strains were performed as described by Walker et al. [31]. Parental strain were cultured at 17°C, in the dark, on MA medium for two weeks and then incubated for one month at 4°C, to induce the formation of microconidia and sclerotia. We mixed three to six sclerotia with 1 ml of microconidia suspension in transparent 24-well microtiter plates. SAS405 and SAS56 are reference strains for MAT1-1 and MAT1-2 mating types, respectively [33]. Cross preparations were stored at 10°C, in 16 h photoperiod conditions, until apothecia developed. Single ascospore-colonies were then isolated by dissecting the apothecia in sterile water and by spreading the ascospores on MM. The ascospore isolates were tested for production of the red pigment on MM, their resistance to fenhexamid and the presence of *bcbik6* and *bcbik1* by using the primer couples Bik1-F6/Bik1-R6 and Bik-F1/Bik-R1 in a diagnostic PCR (Table S1 and Table S3). Sexual recombination among the offspring was assessed by determining the mating-type of each individual using the forward primer MatF and the specific reverse primers Mat1-1R and Mat1-2R (Table S1 and Table S3).

### Functional characterization of BcVEL1 in the pink strain 1750

Recently, *bcvel1* encoding one of the four fungal-specific VELVET proteins of *B. cinerea* was inactivated by a replacement approach in the wild-type strain B05.10 (mutants are in the following designated as B05.10: Δ*bcvel1*) [32]. The same deletion construct comprising a hygromycin resistance cassette that is flanked by 5′ and 3′-noncoding regions of *bcvel1* was used for deletion of *bcvel1* in the pink strain 1750. For that, the replacement construct (3,419 bp) was amplified using primers *bcvel1*-5F and *bcvel1*-3R (Table S1) and transformed into protoplasts of strain 1750 as described previously [53]. Hygromycin-resistant colonies were transferred to agar plates containing Gamborg's B5 medium (Duchefa Biochemie BV, The Netherlands) supplemented with 2% glucose, 0.1% yeast extract and the respective selection agent in a concentration of 70 µg/ml. Single conidial transformants were obtained by spreading conidial suspensions on selective media and transferring single colonies to new plates. Homologous integration events in hygromycin-resistant transformants were detected by diagnostic PCR using the primers pCSN44-trpC-T and pCSN44-trpC-P, binding in the hygromycin resistance cassette and the primers *bcvel1*-hi5F and *bcvel1*-hi3R, binding upstream and downstream of the *bcvel1* flanking regions. Single spore transformants were screened for the absence of the *bcvel1* allele by using primers *bcvel1*-WT-F and *bcvel1*-WT-R matching the substituted coding region of *bcvel1*. In summary, two independent deletion mutants of *bcvel1* (T1 and T8) in the 1750 background (designated as 1750: Δ*bcvel1*) were generated that exhibited the same phenotype. Therefore, the data for one transformant (T8) obviously lacking the *bcvel1* transcript, are presented in Fig. 4. Media for cultivation of *B. cinerea* strains were synthetic complete medium (CM) pH 5 [54], CM pH 7.5 supplemented with 0.01% (w/v) of the pH indicator bromothymol blue for detection of oxalic acid secretion, and modified Czapek Dox (CD) medium pH 5 containing 20 g L^−1^ sucrose, 1 g L^−1^ KH_2_PO_4_, 0.5 g L^−1^ KCl, 0.5 g L^−1^ MgSO_4_ 7H_2_O, and 3 g L^−1^ NaNO_3_ as nitrogen source for detection of melanin formation (CD-M) or 4 g L^−1^ NH_4_NO_3_ as nitrogen source for detection of bikaverin formation (CD-B). Solid media were supplemented with 15 g L^−1^ agar. For studying gene expression by northern blot analyses, the strains were grown for 3 days on CD-B agar with cellophane overlays in continuous darkness at 20°C. Total RNA was isolated making use of the TRIzol reagent (Invitrogen, The Netherlands). Samples (25 µg) of total RNA were transferred to Hybond-N+ membranes after electrophoresis on a 1% (w/v) agarose gel containing formaldehyde, according to the method of Sambrook et al. [50]. Blot hybridizations with random-primed α-^32^P-dCTP-labelled probes were performed as previously described [55].

### Expression *bcbik4* and *bcbik5* in respective *F. fujikuroi ffbik* deletion mutants

For generation of complementation constructs, the open reading frames of *bcbik4* (981 bp) and *bcbik5* (2,366 bp) were amplified from genomic DNA of the gray strain B05.10 using primer pairs *bcbik4*-P*oliC*-F/*bcbik4*-T*gluc*-R and *bcbik5*-P*oliC*-F/*bcbik5*-T*gluc*-R, respectively. Primers comprised extensions facilitating yeast recombinational cloning: thus, the PCR fragments were assembled with plasmid pNDN-OGG [53] digested with *Nco*I and *Not*I, yielding vectors pNDH-OB4G and pNDH-OB5G containing *bcbik4* or *bcbik5* under control of the constitutive *OliC* promoter from *Aspergillus nidulans* and the *gluc* terminator from *B. cinerea*.

The nourseothricin-resistant mutants *F. fujikuroi* Δ*ffbik4* and Δ*ffbik5* [14] were transformed with plasmids pNDH-OB4G and pNDH-OB5G, respectively, mediating the resistance to hygromycin (Table 2). Preparation and transformation of *F. fujikuroi* protoplasts was carried out as previously described [56]. Protoplasts were regenerated at 28°C in regeneration agar (0.7 M sucrose, 0.05% yeast extract, 0.1% casamino acids) supplemented with 100 µg/ml hygromycin B. Random integration of the plasmids containing the *B. cinerea* genes were verified by PCR using primers P*oliC*-sF2 and T*gluc*-sR2 spanning the open reading frames of *bcbik4* and *bcbik5*.

**Table 2:** Fungal strains used in this study for genetic modifications.

Fungus/strain	Description/genotype	Reference
***Botrytis cinerea***		
B05.10	Isolated from *Vitis* (Germany); *MAT1-1*	[59]
B05.10:Δ*bcvel1*	B05.10, Δ*bcvel1::hph*, homokaryon	[32]
1750	Isolated from cucumber (Japan); *MAT1-1*	This study
1750:Δ*bcvel1*	1750, Δ*bcvel1::hph*, homokaryon	This study
***Fusarium fujikuroi***		
IMI58289	Wild-type strain	Commonwealth Mycological Institute, UK
Δ*ffbik4*	IMI58289, Δ*ffbik4::nat1*	[14]
Δ*ffbik4 + bcbik4*	IMI58289, Δ*ffbik4::nat1, OE bcbik4^B05.10^::hph*	This study
Δ*ffbik5*	IMI58289, Δ*ffbik5::nat1*	[14]
Δ*ffbik5+ bcbik5*	IMI58289, Δ*ffbik5::nat1, OE bcbik5^B05.10^::hph*	This study

For submerged cultures, *F. fujikuroi* was pre-incubated at 28°C and 190 rpm for 72 h in 100 ml of Darken medium [57]. For detection of bikaverin production, 0.5 ml of the pre-culture was used to inoculate 100 ml of ICI media [58] containing 6 mM glutamine (10% ICI medium), and incubated at 28°C and 190 rpm for up to five days.

### Virulence tests

Infection assays of *B. cinerea* WT strains and mutants were done by inoculating mycelium or conidia on French bean (*Phaseolus vulgaris*, Caruso cultivar) grown in a climatic chamber. For mycelium test, leaves were harvested from 2 weeks plants and placed in a transparent plastic box lined with tissue moistened with water. Leaves were inoculated with 1.8 mm diameter-plugs of 3 days old mycelium obtained on MA medium. Storage boxes containing inoculated leaves were incubated in a growth cabinet at 21°C with 16 h daylight. Disease development on leaves was recorded daily as radial spread from the inoculation point to the lesion margin. Pathogenicity assays on leaves were repeated three times using at least five leaves per assay. For conidial tests, living *P. vulgaris* plants were used. Conidia were re-suspended in Gamborg's B5 medium supplemented with 2% glucose and 10 mM KH_2_PO_4_/K_2_HPO_4_, pH 6.4. Droplets (7.5 µl) of conidial suspensions (2×10^5^ conidia/ml) were used for inoculation. Plants were incubated in plastic propagator boxes at 20°C under natural illumination and humid conditions.

## Supporting Information

Figure S1 **Comparative phylogenies of BIK1 to BIK6.** Alignments of the protein sequences were performed using the PhyML program [52]. *Maximum likelihood* phylogenies from alignments of the protein sequences were estimated using the PhyML program with the substitution model WAG and a number of bootstraps of 100 [52] (http://www.phylogeny.fr). The last tree is based on the concatenated proteins BIK1 to BIK6. Selected proteins are from the pink *B. cinerea* strain 1750 (EMBL: HE802550, HE802545, HE802546, HE802547, HE802549, HE802548), *F. fujikuroi* (GenBank: CAB92399.1, CAJ75275.1, CAJ75274.1, CAM90598.1, CAM90597.1, CAM90596.1), *F. oxysporum* (BROAD: FOXG_04757.3, FOXG_04756.3, FOXG_04755.3, FOXG_04754.3, FOXG_04753.3, FOXG_04752.3), *F. verticillioides* (BROAD: FVEG_03379.3, FVEG_03380.3, FVEG_03381.3, FVEG_03383.3, FVEG_03382.3, FVEG_03384.3). Protein sequences from *A. niger* (JGI: ANG51499, ANG190154, ANG185884, ANG189113, ANG130183, ANG172695) were used to root the trees.
(TIFF)

Figure S2 **Alignment of** ***bcvel1*** **sequences from the pink strains 1750 and 1787 and the gray strains B05.10 and T4.** Nucleotide sequences were aligned using Lasergene's MegAlign (DNASTAR). SNPs that resulted in stop mutations (T4 and 1750) are indicated in blue, in silent mutations in green (1750 and 1787) and SNPs that lead to an amino acid exchange (1787) are indicated in red. GenBank accession numbers are: *bcvel1* ^B05.10^ (GenBank: HE977589), *bcvel1* ^T4^ (GenBank: HE977590), *bcvel1* ^1750^ (GenBank: HF549030), *bcvel1* ^1787^ (GenBank: HF549031).
(TIFF)

Table S1 **Oligonucleotidic primers used for diagnostic PCR, sequencing, Tail-PCR and cloning.**
(DOCX)

Table S2 **Screening for the presence of** ***BcBIK1*** **and** ***BcBIK6*** **genes in different** ***Botrytis*** **strains using PCR.**
(DOCX)

Table S3 **Phenotypes and genotypes of 1750× SAS405 progeny.**
(DOCX)
