# Supplementary figures and images for: A Functional Bikaverin Biosynthesis Gene Cluster in Rare Strains of Botrytis cinerea Is Positively Controlled by VELVET
Source: PLoS One. 2013 Jan 7;8(1):e53729. doi: 10.1371/journal.pone.0053729 (PMC3538735; doi:10.1371/journal.pone.0053729)

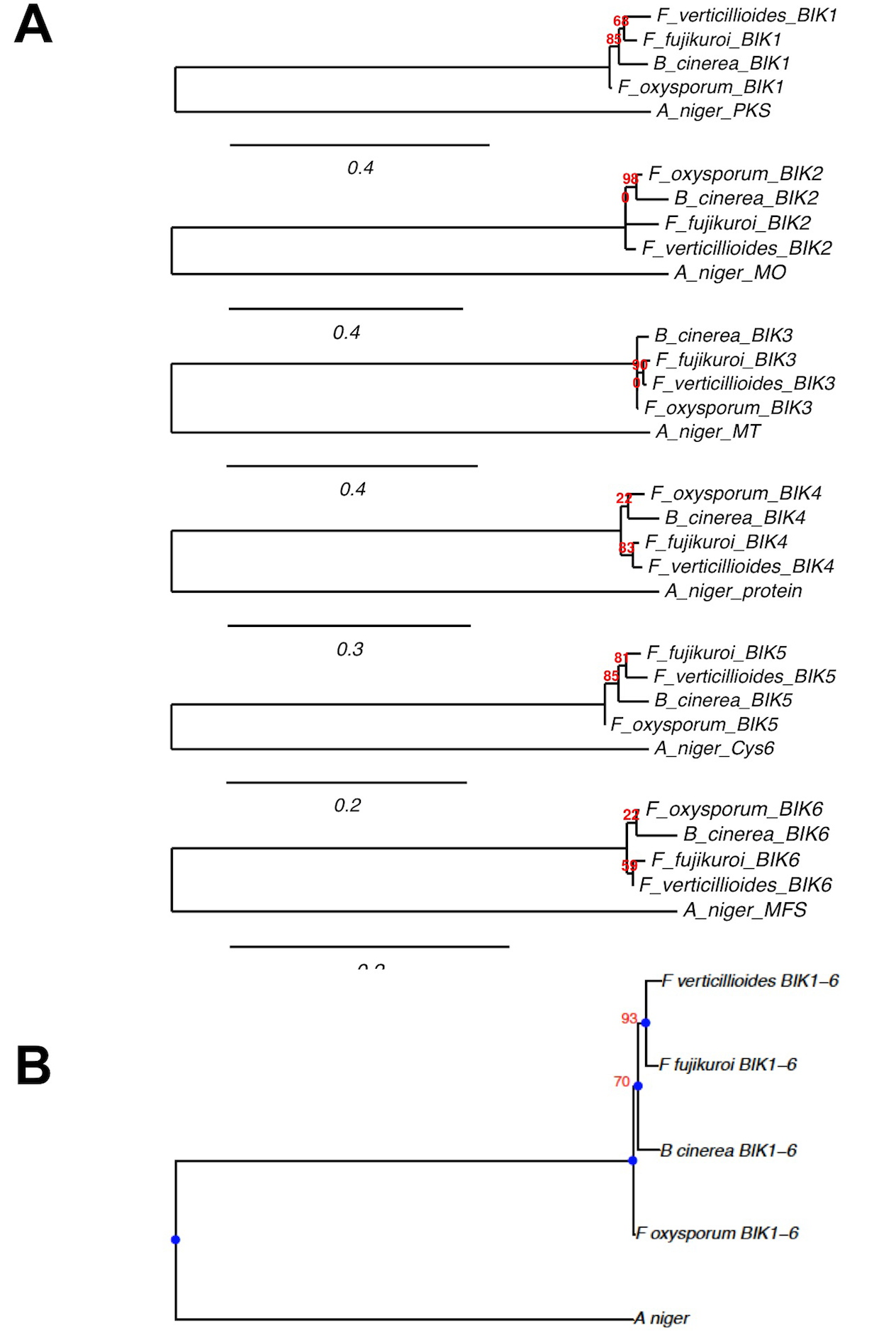

Supplement: Figure S1 — Comparative phylogenies of BIK1 to BIK6. Alignments of the protein sequences were performed using the PhyML program [52]. Maximum likelihood phylogenies from alignments of the protein sequences were estimated using the PhyML program with the substitution model WAG and a number of bootstraps of 100 [52] (http://www.phylogeny.fr). The last tree is based on the concatenated proteins BIK1 to BIK6. Selected proteins are from the pink B. cinerea strain 1750 (EMBL: HE802550, HE802545, HE802546, HE802547, HE802549, HE802548), F. fujikuroi (GenBank: CAB92399.1, CAJ75275.1, CAJ75274.1, CAM90598.1, CAM90597.1, CAM90596.1), F. oxysporum (BROAD: FOXG_04757.3, FOXG_04756.3, FOXG_04755.3, FOXG_04754.3, FOXG_04753.3, FOXG_04752.3), F. verticillioides (BROAD: FVEG_03379.3, FVEG_03380.3, FVEG_03381.3, FVEG_03383.3, FVEG_03382.3, FVEG_03384.3). Protein sequences from A. niger (JGI: ANG51499, ANG190154, ANG185884, ANG189113, ANG130183, ANG172695) were used to root the trees. (TIFF) [file pone.0053729.s001.tiff]

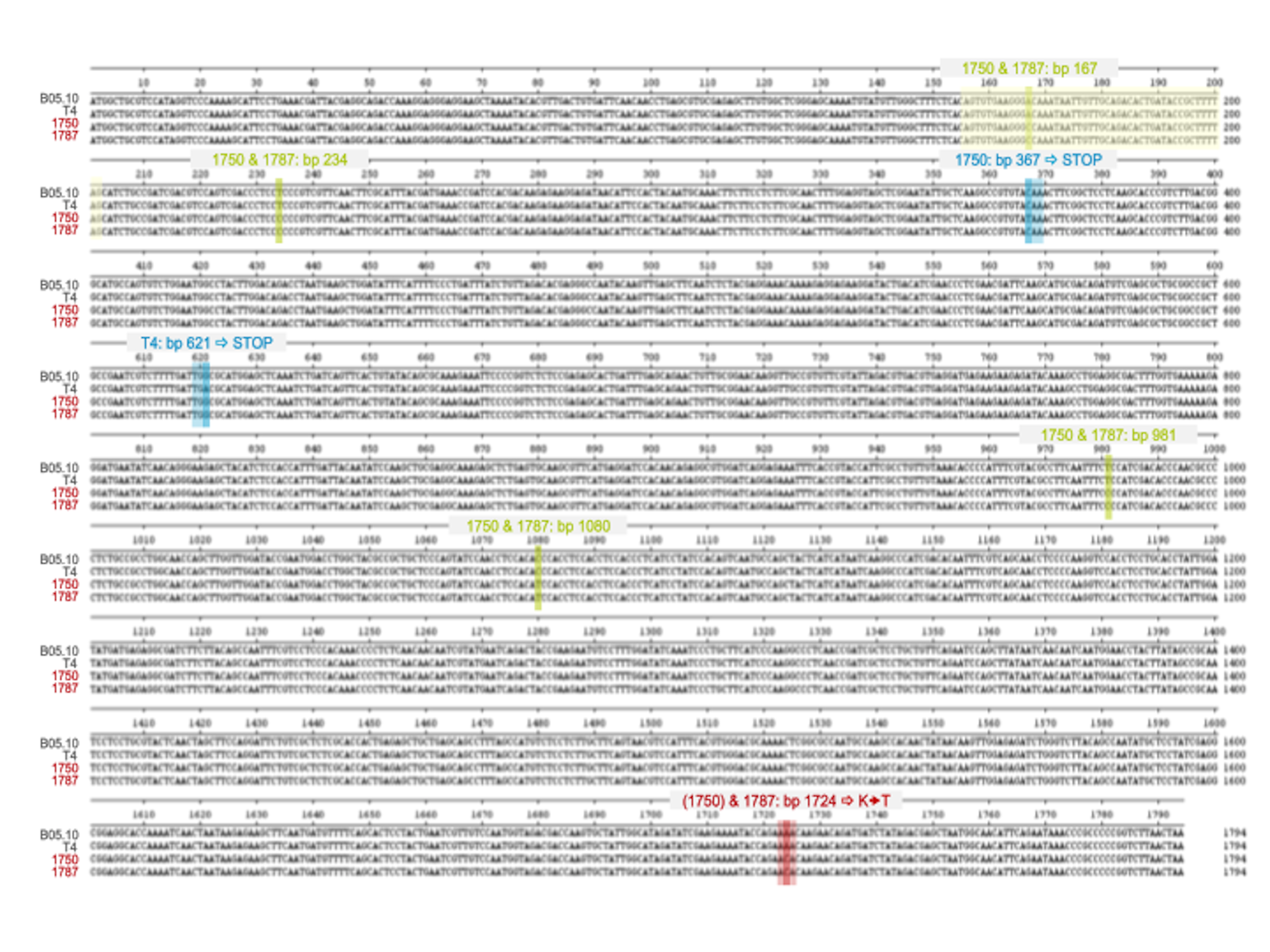

Supplement: Figure S2 — Alignment of bcvel1 sequences from the pink strains 1750 and 1787 and the gray strains B05.10 and T4. Nucleotide sequences were aligned using Lasergene's MegAlign (DNASTAR). SNPs that resulted in stop mutations (T4 and 1750) are indicated in blue, in silent mutations in green (1750 and 1787) and SNPs that lead to an amino acid exchange (1787) are indicated in red. GenBank accession numbers are: bcvel1 B05.10 (GenBank: HE977589), bcvel1 T4 (GenBank: HE977590), bcvel1 1750 (GenBank: HF549030), bcvel1 1787 (GenBank: HF549031). (TIFF) [file pone.0053729.s002.tiff]
