# Supplementary material for: A Functional Bikaverin Biosynthesis Gene Cluster in Rare Strains of Botrytis cinerea Is Positively Controlled by VELVET
Source: PLoS One. 2013 Jan 7;8(1):e53729. doi: 10.1371/journal.pone.0053729 (PMC3538735; doi:10.1371/journal.pone.0053729)
Supplement: Table S1 — Oligonucleotidic primers used for diagnostic PCR, sequencing, Tail-PCR and cloning. (DOCX) [file pone.0053729.s003.docx]

**Table S1:** Oligonucleotidic primers used for diagnostic PCR, sequencing, Tail-PCR and cloning.

| **Primer** | **Sequence (5’ ⭢ 3’)** | **Use** |
| --- | --- | --- |
| **BikA-F** | TCATAAATGACGAAATAGACAACG | 2172 pb fragment for sequencing |
| **BikB-R** | CGAAATTGGCCATTACGAG |  |
| **BikB-F** | TTTCTTGACGCTCGTAATGG | 1400 pb fragment for sequencing |
| **Bik5deb-R** | GACTCTACGTTGGCGACAGTG |  |
| **BikE-F** | TCTCGCAGTATCCAACACCA | 1480 pb fragment for sequencing |
| **BikF-R** | ACCCTCCATCCCTGCTTATC |  |
| **BikG-F** | TGTTTAGGCTCCGAAGATCG | 2920 pb fragment for sequencing |
| **BikH-R** | AAACTTCCCGAGCCTAGCTT |  |
| **BikIbis-F** | CAGGGGTACAAGCTCGAAGAT | 2312 pb fragment for sequencing |
| **BikJter-R** | TCGCAGCCTTCTTACTCTCCTT |  |
| **BikK-F** | CTTGAGAAGTGGTTCTCTTATGAC | 3495 pb fragment for sequencing |
| **BikL-R** | ACTGGACTTGGCTGGTGTTC |  |
| **BikM-F** | GATGCTCATTCCCATCATCTC | 5292 pb fragment for sequencing |
| **BikN-R** | GTGGTTGGCATTCTCAATCG |  |
| ***Bik*-F1** | ggtagcgactagagccgttg | Tail PCR, 1^st^ round |
| ***Bik*-F2** | gcggaataagtttgcgagtc |  |
| ***Bik*-F3** | gcgctgtatttggaggat |  |
| ***Bik1*-F7** | GCACTGTCTCAGCAAATCCA | Tail-PCR, 2^nd^ round |
| ***Bik1*-F8** | CACAGAGACTTGCCCTCCTC |  |
| ***Bik1*-F9** | GTCATGACGAACCCTGACAA |  |
| ***Bik1*-F6** | GTTCGCAGACGAGCTGAAA | Diagnostic PCR for the presence of *BcBIK1* |
| ***Bik1*-R6** | TCGATCTTTGGTCAGGCTCT |  |
| ***Bik*-F1** | ggtagcgactagagccgttg | Diagnostic PCR for the absence of *BcBIK1* |
| ***Bik*-R14** | AGGACGCCGAGACACTAAGA |  |
| ***Bik6*-F2** | AAGATGGTGGTGAAGCCAAC | Diagnostic PCR for the presence of *BcBIK6* |
| ***Bik6*-R1** | ATCTGCCTGCTGCTTACGAT |  |
| ***Mat-F*** | AGCTTCTGTTGGTGCGAAGT | Diagnostic PCR for the determination of the Mating type |
| ***Mat1-1-R*** | GGGCGACTTTTTCAGTCTTC |  |
| ***Mat1-2-R*** | ATGTGGGCTGTTGCAGGTTT |  |
| ***Bcvel1*-F1** | CACCACTCGACACCACCCACTC | Sequencing of *bcvel1* |
| ***Bcvel1*-COM-R** | GCATAAACAACACCCCCGGGAGTCGTATAG | Sequencing of *bcvel1* |
| ***Bcvel1*-F2** | GGTCTCGAGCCGGCCACTGGAAG | Sequencing of *bcvel1* |
| ***Bcvel1*-5F** | GTAACGCCAGGGTTTTCCCAGTCACGACGGTGCCATGTGTGTTGCTGAC | *Bcvel1* deletion construct |
| ***Bcvel1*-3R** | GCGGATAACAATTTCACACAGGAAACAGCACCTTCTACCTCCCATCTAC | *Bcvel1* deletion construct |
| ***Bcvel1*-hi5F** | GAGCGCCATTGGTAGGTAGTCCG | Diagnostic PCR, *bcvel1* deletion |
| **pCSN44-*trpC*-T** | GGAATAGAGTAGATGCCGACCGG | Diagnostic PCR, *bcvel1* deletion |
| ***Bcvel1*-hi3R** | GGCTATTAGCTATTAGCTATTAGTCG | Diagnostic PCR, *bcvel1* deletion |
| **pCSN44-*trpC*-P** | CCTCCACTAGCTCCAGCCAAGCCC | Diagnostic PCR, *bcvel1* deletion |
| ***Bcvel1*-WT-F** | GGGCATGCCAGTGTCTGGAATGG | Diagnostic PCR, *bcvel1* deletion |
| ***Bcvel1*-WT-R** | CTAGTTGAGTACGCAGGAGGATTGC | Diagnostic PCR, *bcvel1* deletion |
| ***Bcbik4*-PoliC-F1** | ccatcacatcacaatcgatccaaccATGTCATCTCCACATTTCTCCAAA | *Bcbik4* complementation vector |
| ***Bcbik4*-Tgluc-R1** | taatcatacatcttatctacatacgTCAGTTGAAGTACCTTGGTATC | *Bcbik4* complementation vector |
| ***Bcbik5*-PoliC-F1** | ccatcacatcacaatcgatccaaccATGGACTCTACTTTGCGAAATCTC | *Bcbik5* complementation vector |
| ***Bcbik5*-Tgluc-R1** | taatcatacatcttatctacatacgTTAATTAAATCTTGGTCGCCCAA | *Bcbik5* complementation/ GFP vector |
| ***Bcbik5*-*gfp*-R1** | tacttacctcacccttggaaaccatATTAAATCTTGGTCGCCCAAGAT | *Bcbik5* GFP vector |
| ***PoliC*-sF2** | GGGAGACGTATTTAGGTGCTAGGG | Diagnostic PCR, complementation approaches |
| **T*glu*c-sR2** | CCGCCCTCTTTTGTCTTCCGC | Diagnostic PCR, complementation approaches |
