# Supplementary material for: A Functional Bikaverin Biosynthesis Gene Cluster in Rare Strains of Botrytis cinerea Is Positively Controlled by VELVET
Source: PLoS One. 2013 Jan 7;8(1):e53729. doi: 10.1371/journal.pone.0053729 (PMC3538735; doi:10.1371/journal.pone.0053729)
Supplement: Table S2 — Screening for the presence of BcBIK1 and BcBIK6 genes in different Botrytis strains using PCR. (DOCX) [file pone.0053729.s004.docx]

**Table S2:** Screening for the presence of *BcBIK1* and *BcBIK6* genes in different *Botrytis* strains using PCR.

| **Species** | **Strain** | **Location and year of isolation** | **Host plant** | **BcBIK6** | **BcBIK1** |
| --- | --- | --- | --- | --- | --- |
| *B. cinerea* | INRA 651 | Bar sur Seine, France (1994) | *Vitis vinifera* | *+* | *-* |
| *B. cinerea* | INRA 704 | Plumecoq, France (1994) | *Vitis vinifera* | *+* | *-* |
| *B. cinerea* | INRA 1476 | Tour Blanche, France (1998) | *Vitis vinifera* | *+* | *-* |
| *B. cinerea* | INRA 1482 | Tour Blanche, France (1998) | *Vitis vinifera* | *+* | *-* |
| *B. cinerea* | INRA 1484 | Tour Blanche, France (1998) | *Vitis vinifera* | *+* | *-* |
| *B. cinerea* | INRA 1486 | Tour Blanche, France (1998) | *Vitis vinifera* | *+* | *-* |
| *B. cinerea* | INRA 1656 | Turckein, France (1998) | *Vitis vinifera* | *+* | *-* |
| *B. cinerea* | INRA 1658 | Turckein, France (1998) | *Vitis vinifera* | *+* | *-* |
| *B. cinerea* | INRA 1730 | Pujols sur Ciron, France (1998) | *Vitis vinifera* | *+* | *-* |
| *B. cinerea* | INRA 1736 | Pujols sur Ciron, France (1998) | *Vitis vinifera* | *+* | *-* |
| *B. cinerea* | INRA 1737 | Pujols sur Ciron, France (1998) | *Vitis vinifera* | *+* | *-* |
| *B. cinerea* | INRA 1738 | Pujols sur Ciron, France (1998) | *Vitis vinifera* | *+* | *-* |
| *B. cinerea* | INRA 1739 | Pujols sur Ciron, France (1998) | *Vitis vinifera* | *+* | *-* |
| *B. cinerea* | INRA 1740 | Pujols sur Ciron, France (1998) | *Vitis vinifera* | *+* | *-* |
| *B. cinerea* | INRA VD 226 | Courteron, France (2007) | *Vitis vinifera* | *+* | *-* |
| *B. cinerea* | Uni. Bari, SAS405 | Italy (<1990) | *Vitis vinifera* | *+* | *-* |
| *B. cinerea* | INRA, UWS111 | Sydney, Australia (2001) | *Vitis vinifera* | + | - |
| *B. cinerea* | Uni. California, TM2413 | North Carolina, USA | *Vitis vinifera* | + | + |
| *B. cinerea* | INRA T4 | Eyragues, France (1991) | *Lycopersicon esculentum* | + | - |
| *B. cinerea* | INRA 2056 | Gent, Belgium (2004) | *Lycopersicon esculentum* | *+* | *-* |
| *B. cinerea* | INRA P462 | France | *Lycopersicon esculentum* | *+* | *-* |
| *B. cinerea* | INRA 2060 | Takelsa, Tunisia (2004) | *Lycopersicon esculentum* | *+* | *-* |
| *B. cinerea* | INRA SEP159 | Avignon, France (<2008) | *Lycopersicon esculentum* | + | + |
| *B. cinerea* | INRA VD390 | Courteron, France (2007) | *Rubus fruticosus* | + | - |
| *B. cinerea* | INRA P1 | Unknown | [*Capsicum annuum*](http://fr.wikipedia.org/wiki/Capsicum_annuum) | *-* | *-* |
| *B. cinerea* | Uni. California, TM517 | USA | *Prunus dulcis* | + | + |
| *B. cinerea* | Uni. California, TM66B02 | USA | *Pistacia vera* | + | + |
| *B. cinerea* | Uni. California, TM2884 | USA | *Rosa spp.* | + | + |
| *B. cinerea* | Uni. Münster, B05.10 | Germany (1994) | Unknown | + | - |
| *B. cinerea* | INRA 1750 | Japan (unknown date) | *Cucumis sativus* | + | + |
| *B. cinerea* | INRA 1787 | Japan (1997) | *Fragaria spp.* | + | + |
| *B. cinerea* | INRA 1789 | Japan (1997) | Unknown | + | - |
| *B. cinerea* | INRA 1790 | Japan (1997) | Unknown | + | - |
| *B. pelargonii* | MUCL1152 | Frederikstad, Norway (1960) | *Pelargonium inquinans* | + | - |
| *B. pelargonii* | CBS497.50 | Frederikstad, Norway (1949) | *Pelargonium inquinans* | *+* | *-* |
| *B. pseudocinerea* | INRA 413 | Isle sur Sorgue, France (1991) | *Lactuca spp.* | + | - |
| *B. pseudocinerea* | INRA 780 | Plumecoq, France (1995) | *Vitis vinifera* | - | - |
| *B. pseudocinerea* | INRA 900 | Boursault, France (1995) | *Vitis vinifera* | + | - |
| *B. pseudocinerea* | INRA VD233 | Courteron, France (2007) | *Vitis vinifera* | + | - |
| *B. pseudocinerea* | INRA VD296 | Courteron, France (2007) | *Vitis vinifera* | + | - |
| *B. pseudocinerea* | INRA VD312 | Courteron, France (2007) | *Vitis vinifera* | + | - |
| *B. fabae* | INRA 2232 | Korba, Tunisia (2004) | *Vicia fabae* | *-* | *-* |
| *B. fabae* | INRA 2220 | Tunis, Tunisia (2004) | *Vicia fabae* | *-* | *-* |
| *B. fabae* | CBS109.57 | Beesd, The Netherlands (1957) | *Vicia fabae* | *-* | *-* |
| *B. fabae* | INRA 2237 | Mateur, Tunisia (2004) | *Vicia fabae* | *-* | *-* |
| *B. fabae* | Arvalis10001 | Maine-et-Loire, France (2010) | *Vicia fabae* | *-* | *-* |
| *B. fabae* | Arvalis11001 | Bazolles, France (2011) | *Vicia fabae* | *-* | *-* |
| *B. calthae* | MUCL2830 | Gatineau Park, Canada (1961) | *Caltha palustris* | *-* | *-* |
| *B. calthae* | MUCL 1089 | Lovenjoel, Belgium (1960) | *Caltha palustris* | *-* | *-* |
| *B. aclada* | MUCL 8415 | Kitzeberg, Germany (1965) | *Allium cepa* | *-* | *-* |
| *B. paenonia* | MUCL16084 | Hacquegnies, Belgium (1970) | *Paeoniae spp.* | *-* | *-* |
| *B. squamosa* | MUCL1107 | California, USA (1923) | *Allium cepa* | *-* | *-* |
| *B. elliptica* | Uni. Wageningen, Be9714 | Elsloo, The Netherlands | *Lilium spp.* | *-* | *-* |
| *B. ranunculi* | CBS 178.63 | Apula, USA (1963) | *Ranunculus abortivus* | *-* | *-* |
| *B. hyacinthi* | MUCL 442 | Breezand, The Netherlands (1958) | *Hyacinthus sp.* | *-* | *-* |
| *B. croci* | MUCL 436 | Breezand, The Netherlands (1968) | *Crocus versicolor* | *-* | *-* |
| *B. tulipae* | Uni. Wageningen, Bt9901 | Tollebeet, The Netherlands (2000) | *Tulipa sp.* | *+* | *-* |
| *B. globosa* | MUCL 444 | Waterloo, Belgium (1958) | *Allium ursinum* | *-* | *-* |
| *B. convulata* | MUCL11595 | USA (1968) | *Iris spp.* | *-* | *-* |
| *B. porri* | MUCL 3234 | Unknown place (1926) | *Allium porrum* | *-* | *-* |
| *S. sclerotiorum* | Uni. Florida, 1980 | Nebraska, USA | *Phaseolus vulgaris* | *-* | *-* |
