# Supplementary material for: A Functional Bikaverin Biosynthesis Gene Cluster in Rare Strains of Botrytis cinerea Is Positively Controlled by VELVET
Source: PLoS One. 2013 Jan 7;8(1):e53729. doi: 10.1371/journal.pone.0053729 (PMC3538735; doi:10.1371/journal.pone.0053729)
Supplement: Table S3 — Phenotypes and genotypes of 1750× SAS405 progeny. (DOCX) [file pone.0053729.s005.docx]

**Table S3:** Phenotypes and genotypes of 1750 x SAS405 progeny

| **Strain^a^** | **Color of the mycelium** | **PCR bik1^b^ F6/R6** | **PCR bik^c^ F1/R14** | **Sensitivity to fenhexamid^d^** | **Mating type**^e^ |
| --- | --- | --- | --- | --- | --- |
| **1750** | **pink** | **+** | **-** | **resistant** | **mat 1-1** |
| **SAS405** | **gray** | **-** | + | **sensitive** | **mat 1-2** |
| 1 | pink | + | - | resistant | mat 1-1 |
| 2 | pink | + | - | resistant | mat 1-1 |
| 3 | pink | + | - | resistant | mat 1-1 |
| 7 | pink | + | - | resistant | mat 1-1 |
| 10 | pink | + | - | resistant | mat 1-1 |
| 14 | pink | + | - | resistant | mat 1-1 |
| 17 | pink | + | - | resistant | mat 1-1 |
| 18 | pink | + | - | resistant | mat 1-1 |
| 19 | pink | + | - | resistant | mat 1-1 |
| 20 | pink | + | - | resistant | mat 1-1 |
| 21 | pink | + | - | resistant | mat 1-1 |
| 22b | pink | + | - | resistant | mat 1-1 |
| 24 | pink | + | - | resistant | mat 1-1 |
| 26 | pink | + | - | resistant | mat 1-1 |
| 27b | pink | + | - | resistant | mat 1-1 |
| 31 | pink | + | - | resistant | mat 1-1 |
| 33 | pink | + | - | resistant | mat 1-1 |
| 36b | pink | + | - | resistant | mat 1-1 |
| 37 | pink | + | - | resistant | mat 1-1 |
| 38 | pink | + | - | resistant | mat 1-1 |
| 39 | pink | + | - | resistant | mat 1-1 |
| 40 | pink | + | - | resistant | mat 1-1 |
| 44 | pink | + | - | resistant | mat 1-1 |
| 56 | pink | + | - | resistant | mat 1-1 |
| 58 | pink | + | - | resistant | mat 1-1 |
| 60 | pink | + | - | resistant | mat 1-1 |
| 63 | pink | + | - | resistant | mat 1-1 |
| 67 | pink | + | - | resistant | mat 1-1 |
| 68 | pink | + | - | resistant | mat 1-1 |
| 72 | pink | + | - | resistant | mat 1-1 |
| 74 | pink | + | - | resistant | mat 1-1 |
| 77 | pink | + | - | resistant | mat 1-1 |
| 78 | pink | + | - | resistant | mat 1-1 |
| 15 | pink | + | - | resistant | mat 1-2 |
| 22a | pink | + | - | resistant | mat 1-2 |
| 23 | pink | + | - | resistant | mat 1-2 |
| 36a | pink | + | - | resistant | mat 1-2 |
| 57 | pink | + | - | resistant | mat 1-2 |
| 64 | pink | + | - | resistant | mat 1-2 |
| 6 | pink | + | - | low resistance | n.a. |
| 12 | gray | - | + | sensitive | mat 1-2 |
| 13 | gray | - | + | sensitive | mat 1-2 |
| 28 | gray | - | + | sensitive | mat 1-2 |
| 34 | gray | - | + | sensitive | mat 1-2 |
| 35 | gray | - | + | sensitive | mat 1-2 |
| 45 | gray | - | + | sensitive | mat 1-2 |
| 46 | gray | n.a. | n.a. | sensitive | mat 1-2 |
| 46 | gray | - | + | sensitive | mat 1-2 |
| 48 | gray | - | + | sensitive | mat 1-2 |
| 54 | gray | - | + | sensitive | mat 1-2 |
| 59 | gray | - | + | sensitive | mat 1-2 |
| 69 | gray | - | + | sensitive | mat 1-2 |
| 70 | gray | - | + | sensitive | mat 1-2 |
| 73 | gray | - | + | sensitive | mat 1-2 |
| 75 | gray | - | + | sensitive | mat 1-2 |
| 80 | gray | - | + | sensitive | mat 1-2 |
| 4 | gray | - | + | sensitive | mat 1-1 |
| 11 | gray | - | + | sensitive | mat 1-1 |
| 27a | gray | - | + | sensitive | mat 1-1 |
| 42 | gray | - | + | sensitive | mat 1-1 |
| 71 | gray | - | + | sensitive | mat 1-1 |
| 76 | gray | - | + | sensitive | mat 1-1 |
| 84 | gray | - | + | sensitive | mat 1-1 |
| 85 | gray | - | + | sensitive | mat 1-1 |
| 8 | gray | - | + | sensitive | n.a. |
| 25 | gray | - | + | sensitive | n.a. |
| 30 | gray | - | + | sensitive | n.a. |
| 53 | gray | - | + | sensitive | n.a. |
| 55 | gray | - | + | sensitive | n.a. |
| 82 | gray | - | + | sensitive | n.a. |
| 83 | gray | - | + | sensitive | n.a. |
| 88 | gray | n.a. | n.a. | sensitive | n.a. |
| 52 | gray | - | + | resistant ^d^ | mat 1-2 |

^a^ Parental strains in bold; ^b, c^ + indicates a PCR product was amplified with primers F6 and R6 (presence of *bcbik1*) or with primers F1 and R14 (absence of *bcbik1*), whereas - indicates that no PCR product was amplified; ^d^ sensitivity to fenhexamid was established as described in Materials and Methods. Resistant strains grew at fenhexamid concentrations up to 5 µgml^-1^. Low resistance corresponds to growth at 0.5 µg ml^-1^ fenhexamid, but no growth at higher concentrations; ^e^ Mating type were determined with specific primers (Table S1); n.a. = not analyzed.
